# Therapeutic management of dexamethasone in patients undergoing endodontics: a randomized clinical trial

**DOI:** 10.1007/s10266-025-01277-0

**Published:** 2025-12-19

**Authors:** Heilyn Joanna Nils, Cristina Arce Recatalá, Cosimo Galletti, Javier Flores-Fraile

**Affiliations:** 1 Department of Stomatology and Surgery, Faculty of Medicine, University of Salamanca, 37008 Salamanca, Spain; 2 Valencia International University (VIU), iBIO Research Group, Valencia, Spain; 3 Faculty of Medicine and Surgery, Kore University of Enna, Enna, Italy; 4 Department of Dental Research Cell, Dr. D.Y. Patil Dental College & Hospital, Dr. D.Y. Patil Vidyapeeth (Deemed to be University), Pimpri, Pune, India

**Keywords:** Dexamethasone, Endodontics, Postoperative pain, Glucocorticoids, Preemptive analgesia, Irreversible pulpitis

## Abstract

Optimal postoperative analgesia remains pivotal in endodontic success, particularly under acute inflammatory conditions. Although glucocorticoids such as dexamethasone exhibit potent anti-inflammatory efficacy, their prophylactic application in endodontics has not been extensively validated. This study assessed whether preoperative dexamethasone offers superior analgesia to postoperative ibuprofen in endodontic pain control. A randomized, controlled Phase IV clinical trial was undertaken at the Faculty of Dentistry, University of Salamanca, involving 82 ASA I patients undergoing single-visit root canal treatment. Subjects were equally randomized into two arms: the experimental group received 4 mg oral dexamethasone one hour preoperatively, while the control group was administered 400 mg ibuprofen at four-hour intervals post-treatment. Postoperative pain was evaluated using the Visual Analog Scale (VAS) at 4, 6, 8, 12, and 24 h. Intergroup differences were analyzed using the Mann–Whitney U test. The dexamethasone cohort demonstrated statistically significant reductions in VAS scores between 6- and 24-h post-intervention (p < 0.001), with no adverse events or need for rescue analgesia observed. Gender-based variation in pain perception was not significant. A single 4 mg preoperative oral dose of dexamethasone markedly enhances early postoperative analgesia in root canal treatment relative to standard ibuprofen regimens. These results support its integration into preemptive root canal protocols, contingent upon further multicenter validation. Approved by the Bioethics Committee (Ref. 2025_01/345; March 10, 2025). Clinical trial registration: NCT069063150 (April 1, 2025).The study adhered to the ethical standards of the 1964 Declaration of Helsinki and its later amendments.

**Graphical abstract:**

**Figure:**
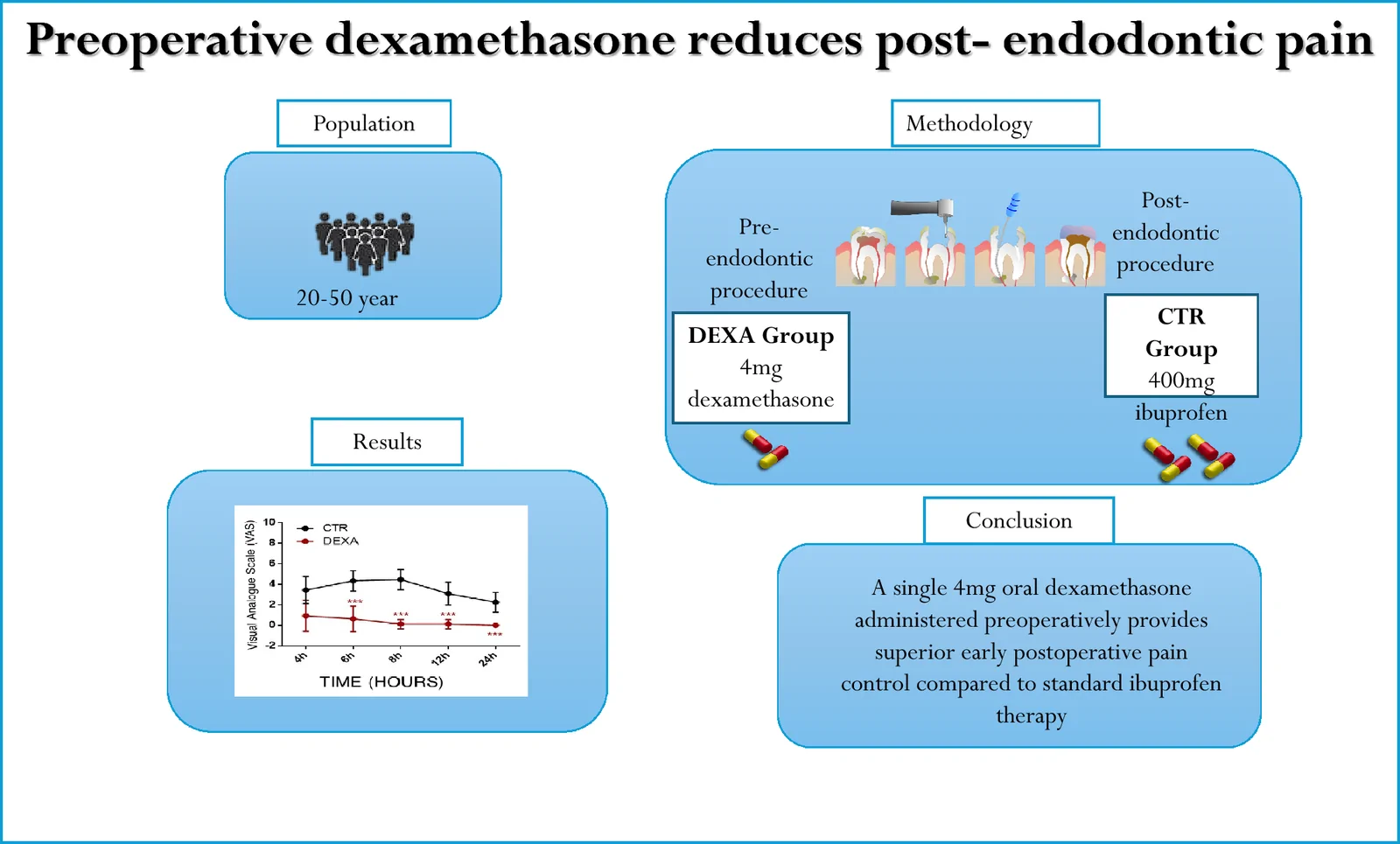

**Supplementary Information:**

The online version contains supplementary material available at https://doi.org/10.1007/s10266-025-01277-0.

## Introduction

Odontogenic pain represents one of the primary motivators driving individuals to seek dental care, rendering a comprehensive understanding of its etiology and clinical presentation essential for accurate diagnosis and effective therapeutic intervention. The oral cavity, characterized by its dense neural network, is highly sensitive, rendering it particularly susceptible to nociceptive stimuli. Dental pain may arise spontaneously, be self-provoked, or be elicited iatrogenically during non-invasive procedures, including root canal treatment [1].

According to the International Association for the Study of Pain (IASP), pain is defined as “an unpleasant sensory and emotional experience associated with actual or potential tissue damage or described in terms of such damage [2].” The perception of pain is mediated by a complex neurophysiological process involving a specialized sensory system composed of nociceptors peripheral receptors specifically responsive to noxious stimuli and the corresponding afferent neural pathways. These components collectively detect, transmit, and process nociceptive signals originating from tissue insult, thereby contributing to the multifaceted sensory and affective dimensions of the pain experience.

Inflammation constitutes a fundamental defensive mechanism of the immune system, initiated in response to cellular injury or tissue insult. This complex biological process may be triggered by a variety of etiological factors, including physical trauma, chemical irritants, thermal extremes, immunological reactions, vascular disturbances, and microbial pathogens[2]. Irrespective of the inciting agent, the ensuing inflammatory cascade characteristically manifests through cardinal clinical signs- (pain), (heat), (redness), and (swelling) as described in classical pathology. Empirical evidence from multiple studies indicates that the inflammatory response typically reaches its peak intensity within 48 to 72 h following the initial insult [3].

Pharmacotherapeutic management within endodontic practice has garnered considerable scholarly attention in recent years, owing to the complex clinical scenarios routinely encountered by endodontists. Conditions such as irreversible pulpitis, pulpal necrosis, and endodontic infections frequently precipitate significant inflammation and pain, necessitating effective analgesic intervention. A wide array of strategies has been explored to mitigate postoperative discomfort following root canal treatment. These include the use of opioid and non-opioid analgesics, intra-canal and systemic corticosteroids, nonsteroidal anti-inflammatory drugs (NSAIDs), long-acting local anesthetics, antibiotics, trephination, occlusal adjustment, and even non-pharmacological modalities such as hypnosis. Among these options, NSAIDs and corticosteroids either administered individually or in combination are the most prevalently utilized agents for the management of post-endodontic pain. Contemporary literature underscores the importance of evaluating pain intensity through standardized assessment tools, with particular emphasis on the World Health Organization’s (WHO) analgesic ladder. This hierarchical model provides a framework for the rational selection of analgesic agents based on pain severity, often quantified via the Visual Analogue Scale (VAS), typically represented by a 10-cm metric ruler. In this context, VAS scores of 1–4 are classified as "mild pain," generally managed with non-opioid analgesics; scores ranging from 5–7 signify "moderate pain," for which weak opioids, either as monotherapy or in conjunction with non-opioids and/or adjuvant agents are recommended; and scores of 8–10 denote "severe pain," warranting the administration of strong opioids, alone or in combination with adjunctive pharmacological therapies [4].

Contemporary dental practice increasingly emphasizes advanced paradigms of pain management, among which the implementation of preventive strategies has emerged as a pivotal approach in mitigating postoperative discomfort [5]. "Preventive therapy" refers to the preemptive administration of pharmacologic agents or the execution of specific clinical interventions aimed at attenuating or inhibiting the nociceptive response prior to the onset of surgical stimuli. The overarching objective of such prophylactic modalities is to forestall the development and persistence of postoperative pain, thereby improving patient comfort and facilitating a smoother recovery trajectory from the immediate conclusion of the procedure to several weeks post-intervention [6]. This anticipatory approach to analgesia represents a significant evolution from traditional reactive models, aligning with evidence-based strategies for optimizing clinical outcomes in surgical and endodontic contexts.

The administration of glucocorticoid-based pharmacotherapy within the scope of endodontic practice represents a viable and efficacious therapeutic alternative, particularly in cases where patients present no contraindications to their short-term or single-dose use. Owing to their potent anti-inflammatory and immunosuppressive properties, glucocorticoids can modulate key pathophysiological mechanisms underlying postoperative inflammation and pain, thereby enhancing patient comfort and clinical outcomes following endodontic procedures [7]. Dexamethasone is a potent synthetic glucocorticoid, structurally derived from endogenous corticosteroids produced by the adrenal cortex under the regulatory influence of adrenocorticotropic hormone (ACTH) secreted by the anterior pituitary. Its principal pharmacological actions include the attenuation of inflammation, suppression of nociceptive signaling, and modulation of the immune response. Traditionally, the administration of local anesthetics has served as the primary modality for managing postoperative pain in dental procedures. However, emerging evidence supports the superior efficacy of preemptive pharmacologic interventions in attenuating postoperative discomfort. Alcántara et al. demonstrated that the preoperative administration of 8 mg dexamethasone in third molar surgeries provided significantly enhanced control over pain and inflammation during the initial 48-h postoperative period when compared to a regimen of 50 mg diclofenac sodium combined with 50 mg codeine phosphate [8].

The theoretical framework underpinning the use of preemptive analgesics and anti-inflammatory agents is centered on their ability to inhibit peripheral sensitization, thereby extending the duration and efficacy of analgesia and inflammatory control. A single pre-procedural dose of a potent anti-inflammatory agent such as dexamethasone promotes the early release of endogenous anti-inflammatory mediators, facilitating modulation of the acute inflammatory response while simultaneously minimizing the risk of adverse effects commonly associated with sustained or repeated dosing schedules.

As a result, the preoperative use of dexamethasone is gaining increased clinical acceptance, particularly given its demonstrated effectiveness in managing odontogenic pain. Moreover, dexamethasone exhibits a favorable safety profile when administered in appropriate, individualized dosages. Adverse effects are predominantly associated with long-term corticosteroid therapy involving high systemic doses. In contrast, intermittent or single-dose administration over a short duration has not been shown to elicit significant harmful effects in otherwise healthy patients [9, 10]. Therefore, careful patient selection and personalized dosing protocols remain essential to optimize therapeutic outcomes while preserving patient safety.

Despite growing interest in the preoperative use of dexamethasone within endodontic practice, an optimal reduced dosage capable of achieving effective analgesic and anti-inflammatory outcomes has yet to be clearly established. In light of this gap. Our hypothesis is that a single preoperative oral dose of 4 mg dexamethasone contributes to a significant decrease in periapical inflammatory response and postoperative pain following root canal treatment.

Accordingly, the primary objective of the present study was to assess the anti-inflammatory and analgesic efficacy of a single preoperative oral dose of 4 mg dexamethasone in controlling postoperative pain following root canal treatment. This evaluation encompassed treatments performed in both maxillary and mandibular teeth, across all dental groups, and was limited to single-visit root canal treatment.

## Materials and methods

This randomized clinical trial (RCT) received ethical approval from the Bioethics Committee of Alfonso X el Sabio University, Madrid, Spain, on March 10, 2025 (*File No. 2025_01/345*), and was duly registered with the ClinicalTrials.gov database (*Identifier: NCT06906315*). The study was conducted in strict adherence to the CONSORT (Consolidated Standards of Reporting Trials) guidelines at the Department of Endodontics, Doctoral Program, Faculty of Dentistry, University of Salamanca, over a three-month period from November 2024 to January 2025. The study was conducted in full accordance with the ethical principles of the Declaration of Helsinki and Good Clinical Practice (GCP) guidelines. Informed consent and data authorization were obtained from all participants prior to enrollment.

### Sample size

The study sample was randomized using a simple random probability sampling method to ensure unbiased allocation and enhance the internal validity of the trial. To optimize the study design and minimize the number of participants required while maintaining adequate statistical power, a formal sample size calculation was performed. The sample size was estimated based on an F-test for two-way analysis of variance (ANOVA), assuming a two-tailed significance level (α) of 0.05, a statistical power of 80% (1 − β), and a confidence level of 95%. Due to the absence of prior data regarding population variability (σ) and the minimum clinically significant difference (δ), an estimated medium effect size of 0.5 was selected in accordance with Cohen’s conventions. This methodological approach provided a statistically sound framework for determining the minimum number of subjects necessary to detect meaningful differences between groups.

A total of 82 patients requiring root canal treatment were enrolled, comprising 31 female and 51 male participants. Subjects were randomly allocated into two equal groups of 41 patients each. The control group received 400 mg of ibuprofen administered every four hours following root canal treatment, while the experimental group was administered a single 4 mg oral dose of dexamethasone precisely one hour prior to the root canal treatment.

Post-treatment, all participants were instructed to self-report their pain intensity using a Visual Analogue Scale (VAS) at predefined intervals: 4-, 6-, 8-, 12-, and 24-h post-procedure. The clinical setting offered comprehensive endodontic care, encompassing advanced procedures such as non-surgical root canal treatment, post removal, regenerative pulp therapies, and endodontic microsurgeries.

A comprehensive anamnesis was conducted for each participant, and a detailed clinical history (CH) was systematically recorded. The clinical history encompassed a range of demographic and medical variables, including age, sex, current pharmacological treatments, existing systemic pathologies, known allergies, and the specific endodontic diagnosis such as irreversible pulpitis (with or without periapical involvement), pulp necrosis, retreatment cases, and endodontic surgical indications.

Prior to initiating root canal treatment, radiographic assessments were performed to evaluate pulpal status and confirm the vitality or necrosis of the dental tissues. In order to ensure a homogeneous and medically stable study population, participants were classified according to the American Society of Anesthesiologists (ASA) physical status classification system [11]. Only ASA Grade I individuals deemed to be in optimal systemic health and free from significant comorbidities were selected for inclusion in this clinical trial.

### Inclusion criteria

1. Patients in need of a root canal treatment.
2. All groups of teeth were eligible (anterior, premolar, molar, both maxillary and mandibular).
3. Patients in the range of 20–50 years of both sexes.

### Exclusion criteria

1. Patients under 20 years of age and over 50 years of age were excluded.
2. Patients who did not agree to take part in the trial.
3. Patients with dental hypersensitivity.
4. Pregnant or breast-feeding women.
5. Patients who have been administered NSAIDs 48 h prior to the intervention.
6. Barbiturate user and immunocompromised patients, tuberculosis, patients with possible systemic fungal infection.
7. Patients with ulcerative colitis, peptide ulcers, diverticulitis, osteoporosis, glaucoma, cataracts, diabetes mellitus, acute psychosis.
8. Tooth with fractured roots.

Prior to participation, all individuals enrolled in the study provided written informed consent. The consent process included a thorough explanation of the research objectives, as well as a clear outline of the potential benefits and risks associated with the preoperative administration of dexamethasone in root canal treatment. Participants were given the opportunity to ask questions and were assured of their right to withdraw from the study at any time without prejudice to their standard care.

### Interventions

Participants in the experimental group received a single oral dose of 4 mg dexamethasone administered precisely one hour prior to the initiation of the root canal treatment. A contingency plan for rescue analgesia was established, to be implemented in instances where adequate pain relief was not achieved within the subsequent 24 h. This measure was deemed essential due to the anticipated interindividual variability in pharmacodynamic response to corticosteroid therapy. Conversely, the control group did not receive any pharmacological intervention prior to the procedure. Upon completion of the root canal treatment, these patients were prescribed the standard postoperative analgesic regimen consisting of 400 mg of ibuprofen administered every four hours, as clinically indicated.

All root canal treatments were performed under strict aseptic and antiseptic conditions to ensure optimal infection control. Local anesthesia was administered in accordance with standard clinical protocols, utilizing 2% lidocaine and/or 4% articaine with epinephrine. A maximum of 2 to 4 carpules of anesthetic solution was permitted, depending on the clinical complexity and patient-specific requirements. Following anesthetic induction, an initial preoperative radiograph was obtained to assess root morphology and guide treatment planning. The operative field was then isolated using a rubber dam to maintain a sterile environment and prevent contamination. A fixed dental operating microscope was employed to enhance visualization and precision throughout the procedure.

Access to the pulp chamber was achieved using a round carbide bur (e.g., #2 #4 #6 initial penetration through enamel/dentin; size depends on tooth anatomy); transmetal bur (if crown present)1557 or 557 (carbide fissure bur), Endo-Z Bur 28 mm length (Dentsply Maillefer Endo-Z Bur) and ultrasonic instruments #1, # 3, 4 (Dentsply Sirona or Acteon). The procedure involved coronal access preparation, removal of the pulp chamber roof and pulp horns, and establishment of straight-line access to the root canal system. The working length of each canal was subsequently determined with the aid of an electronic apex locator to ensure accuracy in canal debridement and obturation.

Instrumentation of the root canals was performed using two established nickel-titanium rotary systems Mtwo (VDW, Munich, Germany) and ProTaper (Dentsply Tulsa Dental Specialties, Tulsa, OK) employing the apex-crown technique to facilitate efficient coronal flaring and apical progression. Initial canal negotiation was achieved manually using stainless steel K-files in sizes 06, 08, 10, or 15, 20 with lengths of 21 mm, 25 mm, or 31 mm, selected based on the anatomical and morphological characteristics of each tooth.

Following glide path establishment, rotary instrumentation was advanced, with the objective of preparing the canal system to a final apical size of ISO 25 or 30, with a taper of approximately 0.04 or 0.06. This preparation allowed for adequate shaping of the canal while preserving apical integrity, facilitating subsequent irrigation, disinfection, and obturation procedures.

In accordance with the clinical requirements for effective cleaning, disinfection, and shaping of the root canal system, chemomechanical debridement was achieved through copious irrigation using a 5.25% sodium hypochlorite (NaOCl) solution. Irrigation was performed throughout the instrumentation process using 27-gauge needles, carefully introduced into the canal with a controlled coronal-to-apical movement. Moderate manual pressure was applied to the syringe to ensure effective delivery of the irrigant while minimizing the risk of apical extrusion.

In selected cases, irrigation with 0.12% chlorhexidine digluconate was employed as an adjunctive irrigant and neutralizing rinse, primarily to take advantage of its substantivity and its ability to reduce residual microbial load without the potential for increased cytotoxicity or undesirable interactions when NaOCl has already been used. This lower concentration is also widely accepted in clinical endodontic protocols when chlorhexidine is used as a final rinse or auxiliary irrigant rather than as the main antimicrobial agent.

Subsequent to the completion of mechanical instrumentation, a 17% ethylenediaminetetraacetic acid (EDTA) chelating solution was introduced into the canal system and allowed to act for one minute. This solution functioned as a lubricant and served to effectively dissolve and eliminate the smear layer and dentinal debris generated during canal preparation. The final irrigation was performed using sterile distilled water or isotonic physiological saline, with the objective of neutralizing and removing residual chemical agents previously employed during the shaping and disinfection phases of the root canal treatment.

Following final irrigation and neutralization, the root canals were meticulously dried using sterile absorbent paper points to ensure complete removal of residual moisture. Obturation was performed using a modified single-cone technique with gutta-percha cones corresponding to the final apical preparation size (0.04/0.06 taper), ensuring optimal adaptation to the canal walls. A biocompatible endodontic sealer either Bio-C Sealer (Angelus, Londrina, Brazil), a calcium silicate-based material, or AH Plus (Dentsply, Tulsa Dental Specialties, Tulsa, OK, USA), an epoxy resin-based sealer was employed to achieve a hermetic seal, enhance apical closure, and prevent reinfection of the root canal system.

Following cone placement, the excess gutta-percha was carefully trimmed to the canal orifice level, and a light lateral condensation technique was applied to enhance the compaction and three-dimensional filling of the root canal treatment. This step ensured complete obturation, particularly in irregular canal anatomies and lateral extensions. The pulp chamber floor was subsequently sealed using a resin-based restorative material or, in cases requiring delayed definitive restoration, a temporary filling was placed using Cavit™ (3 M Deutschland GmbH, Neuss, Germany). Final verification of occlusion was performed to ensure the absence of any high contact points, thereby preventing postoperative discomfort or functional interference.

### Evidence collection instruments

Comprehensive diagnostic procedures were undertaken prior to the initiation of the root canal treatment to ensure precise case selection and the formulation of an appropriate treatment strategy. Pulp vitality was evaluated using thermal sensitivity tests, which involved the application of cold or heat stimuli to the tooth surface to provoke a sensory response from the dental pulp. The patient’s subjective reaction to these stimuli was carefully documented to assess the functional integrity of the pulp tissue, thereby aiding in the differentiation between vital, partially necrotic, and non-vital pulpal states. In addition to thermal testing, a thorough radiographic assessment was conducted. This included periapical radiographs, panoramic imaging, and, when clinically indicated, three-dimensional cone-beam computed tomography (CBCT) scans to assess root morphology, detect periapical pathology, and determine the extent of pulpal and perirradicular involvement.

Further diagnostic evaluations, including percussion, palpation, and occlusal analysis, were performed to identify clinical signs suggestive of periapical periodontitis or other associated perirradicular pathologies. The integration of clinical findings with advanced imaging modalities provided a robust diagnostic foundation for guiding the subsequent root canal treatment.

Assessment of post-endodontic pain: Given the inherently subjective nature of pain perception and the considerable inter-individual variability in pain thresholds, the use of unidimensional pain assessment tools such as the Visual Analogue Scale (VAS) is widely endorsed for quantifying pain intensity in clinical research. Accordingly, this study employed a Likert-type VAS to evaluate postoperative pain following root canal treatment. The VAS utilized consisted of a 10-cm horizontal line, anchored at either end with verbal descriptors representing the extremes of pain experience: "0" on the far left signified the complete absence of pain, while "10" on the far right represented the most severe pain imaginable [12]. Prior to data collection, all participants were thoroughly instructed in the purpose, structure, and interpretation of the VAS instrument to ensure accurate and consistent self-reporting. Patients received verbal and visual training on how to correctly mark the scale and were encouraged to ask questions to clarify any uncertainties. Pain intensity was self-reported by each participant at defined postoperative intervals: 4, 6, 8, 12, and 24 h following the root canal treatment. At each point, patients were asked to select the appropriate numerical value corresponding to their perceived level of pain, encircling the number on the provided survey sheet. Additionally, participants were given the opportunity to record any unscheduled episodes of pain or notable sensory experiences outside the predefined intervals, thereby allowing for a more comprehensive understanding of their postoperative course.

On the seventh postoperative day, patients were scheduled to return to the clinic to submit their completed VAS assessments and initiate the data analysis phase. For participants who reported significant discomfort or inadequate pain control, provisions were made to ensure prompt clinical management. These included expedited in-person appointments, direct transmission of prescriptions to affiliated pharmacies, and follow-up telephone consultations to monitor the patient’s post-treatment recovery and ensure their well-being.

To enhance accessibility and maximize response rates, an electronic submission option for the pain assessment questionnaire was also provided. This alternative was particularly valuable for patients unable to return to the clinic due to occupational, personal, or logistical constraints. The inclusion of digital access ensured comprehensive data collection and supported continuity of care throughout the follow-up period.

### Analysis of data

Data analysis and interpretation were conducted using the Statistical Package for the Social Sciences (SPSS), version 22.0 (IBM Corp., Armonk, NY, USA) by an independent individual not affiliated with the research team. Qualitative variables were described using means, standard deviations and percentages of each category. The Shapiro–Wilk test was applied to assess the normality of the distribution of continuous variables. Mann–Whitney U test was employed to evaluate differences between control and treated groups. All statistical tests were conducted with a 95% confidence level (p < 0.05 considered statistically significant). The analysis adhered to the intention-to-treat principle, ensuring that all randomized participants were included in the group to which they were originally assigned, regardless of adherence to the intervention protocol.

## Results

**Fig. 1:**
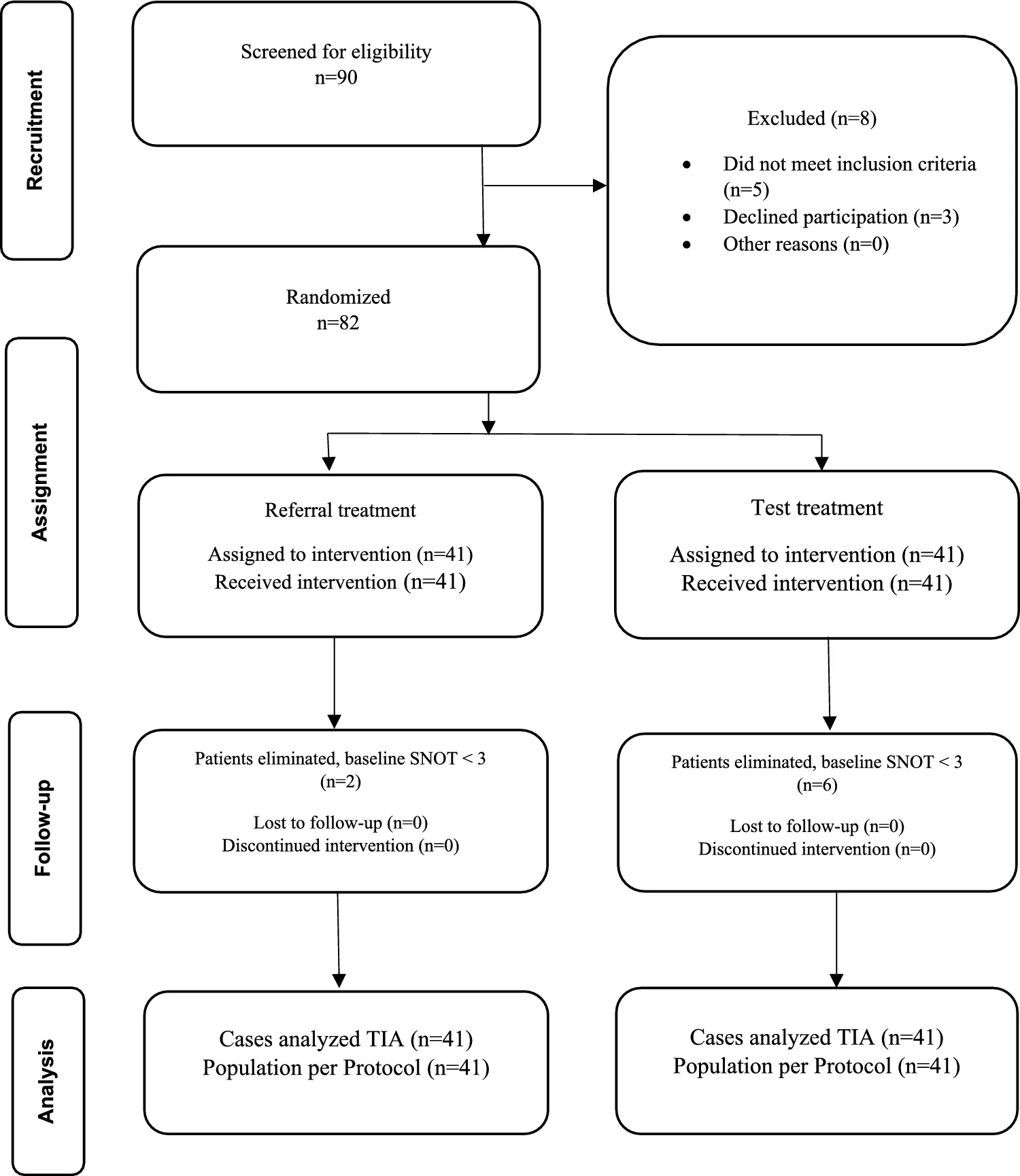
CONSORT flowchart. It reflects the patient selection process

The CONSORT flow diagram (Fig. 1) outlines the sequential process of participant recruitment, allocation, follow-up, and analysis for the present study. Initially, 90 patients were assessed for eligibility. Of these, 8 patients were excluded: 5 individuals did not meet the predefined inclusion criteria, and 3 declined to participate. No patients were excluded for other or unspecified reasons.

Following the eligibility screening, 82 participants were successfully enrolled and randomly assigned into two groups for the intervention phase, with 41 individuals allocated to each arm: the experimental group (preoperative dexamethasone) and the control group (postoperative ibuprofen).During the follow-up phase, a total of 5 patients were excluded from the final analysis due to presenting a baseline Sino-Nasal Outcome Test (SNOT) score below 3, which fell outside the established threshold for inclusion in outcome evaluation. The SNOT is a validated, patient-reported outcome measure widely employed in rhinology to quantify the burden of sinonasal conditions particularly chronic rhinosinusitis on quality of life. The SNOT-22, the most frequently used version, consists of 22 items assessing both physical symptoms (e.g., nasal obstruction, rhinorrhea) and psychosocial impacts (e.g., sleep quality, emotional distress) using a Likert-type scale. Specifically, two participants were excluded from the experimental (dexamethasone) group, and three were excluded from the control (ibuprofen) group. The remaining participants proceeded through the full intervention and were included in the final data analysis set.

The mean age of participants in the dexamethasone group was 33.63 years, with 48.0% identified as female. In the ibuprofen control group, the mean age was 36.78 years, with a slightly higher female representation at 54.84%. No statistically significant differences were observed between groups in terms of age (p = 0.1061), gender distribution (p = 0.989), or baseline diagnosis.

No participants exhibited non-adherence to the assigned pharmacological regimen. Patients with systemic health conditions, known hypersensitivity to NSAIDs and/or corticosteroids, pregnant or lactating individuals, and those with previously initiated or completed root canal treatments were excluded from the study.

### Comparative analysis of preoperative dexamethasone and postoperative ibuprofen on pain intensity following endodontic treatment: a gender-inclusive evaluation using the VAS scale

The results, depicted in (Table 1), illustrate clear differences in pain perception between the two groups over the 24-h evaluation period. The DEXA group consistently reported low pain levels, with VAS scores ranging predominantly between 0 and 2, indicating no to mild discomfort throughout the first 24-h post-procedure. In contrast, the CTR group exhibited higher pain intensity levels, with VAS scores ranging from 2 to 4, corresponding to mild to moderate pain during the same postoperative period. At the 4-h time point, participants in the ibuprofen control group reported a mean VAS score of 3.4, which falls within the moderate pain category. These findings suggest that preoperative administration of dexamethasone may be more effective in attenuating early postoperative pain following root canal treatment.

**Table 1:** Socio-Demographic and Baseline Clinical Characteristics of Participants Stratified by Treatment Group (DEXA vs. CTR). Participants were stratified into two groups: the control group (CTR), comprising individuals who received 400 mg of ibuprofen postoperatively, and the experimental group (DEXA), consisting of individuals who received a single 4 mg oral dose of dexamethasone preoperatively. Descriptive statistics are reported as mean ± standard deviation (SD) for continuous variables and percentages for categorical variables. Intergroup comparisons were conducted using the Mann–Whitney U test. Statistical significance is denoted as s (significant) and ns (not significant)

Variable	CTR (N = 41)	DEXA(N = 41)	p value
Age (mean years ± SD)	36.78 ± 8.51	33.63 ± 9.22	0.1061 ns
Gender (%)			
Female	17 (41.46)	14 (58.54)	0.989 ns
Male	24 (34.14)	27 (63.41)	
Diagnosis (%)			
Irreversible pulpitis	20 (48.78)	20 (48.78)	
Irreversible pulpitis with apical lesion	10 (24.39)	10 (24.39)	
Necrosis	2 (4.88)	2 (4.88)	
Retreatment with periapical lesion	7 (17.07)	7 (17.07)	
Endodontic surgery	2 (4.88)	2 (4.88)	

In contrast, participants in the dexamethasone group reported a mean VAS score of 1.3 at the 4-h mark, indicative of mild pain. This lower pain perception suggests that dexamethasone may exert a preemptive analgesic effect, likely through the inhibition of peripheral sensitization mechanisms. However, it is important to consider that pain scores recorded at the 4-h time point may be partially influenced by the residual effect of local anesthetics administered during the root canal treatment.

**Fig. 2:**
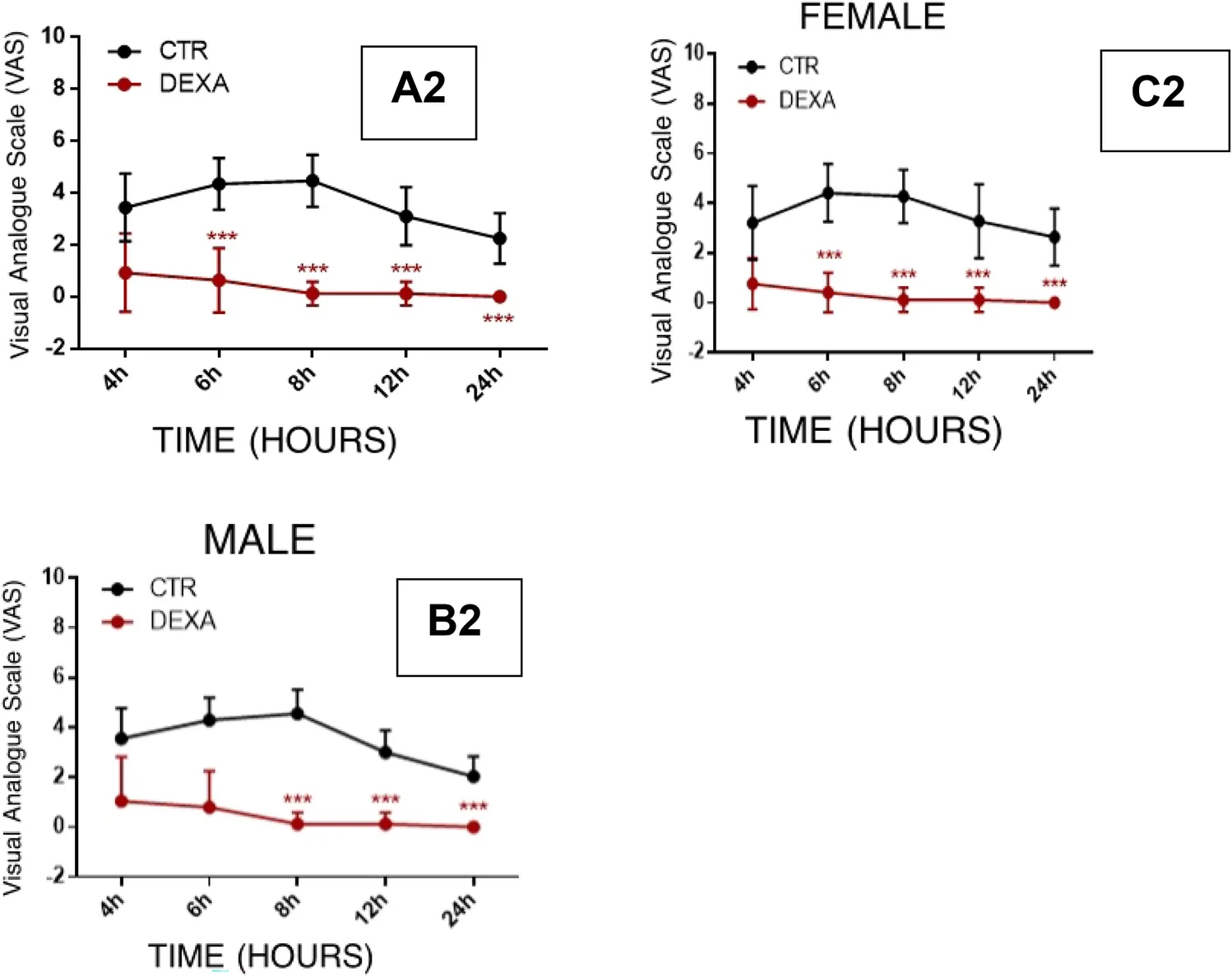
Postoperative Pain Intensity Measured by Visual Analog Scale (VAS) in Dexamethasone (DEXA) and Control (CTR) Groups. **A2** Visual analog scale (VAS) of pain perception as a function of time 4, 6, 8, 12, 24-h post-endodontics in Dexamethasone group (DEXA) compared to the control group (CTR). **B2** VAS in Male. **C2** VAS in female. Statistics: Mean and standard error of pain intensity values for each group at each time point. Significant differences between the groups studied. Statistical analysis ***P = 0.0001 Mann–Whitney test, n = 41 each group

Statistical analysis revealed significant differences in pain intensity between the two groups across all measured time points (4, 6, 8, 12, and 24 h), as illustrated in (Fig. 2). These findings support the hypothesis that preoperative administration of 4 mg dexamethasone is more effective than postoperative administration of 400 mg ibuprofen in controlling pain and inflammation associated with root canal treatment.

Furthermore, subgroup analysis based on gender demonstrated a consistent trend: both male and female participants who received dexamethasone reported lower pain scores compared to those in the ibuprofen group, particularly at the 8-, 12-, and 24-h evaluations. This observation suggests that the analgesic efficacy of preoperative dexamethasone is not influenced by gender and may offer reliable pain control across diverse patient populations.

The dexamethasone group demonstrated significantly lower postoperative VAS pain scores at all evaluated time points compared with the ibuprofen group, indicating superior and more sustained analgesic effectiveness.

## Discussion

The outcomes of this investigation clearly indicate that: (1) administering dexamethasone preoperatively results in substantially greater reduction of postoperative pain after root canal treatment compared with postoperative ibuprofen, and (2) no participants in either group required rescue analgesia, highlighting the adequate pain-control capacity of both medications within the studied parameters.

Clinical diagnoses included symptomatic irreversible pulpitis (with or without associated apical pathology), pulpal necrosis, retreatment cases with periapical involvement, and surgical endodontic indications. Diagnoses were confirmed through comprehensive clinical and radiographic examination. No participants exhibited non-adherence to the assigned pharmacological regimen. Patients with systemic health conditions, known hypersensitivity to NSAIDs and/or corticosteroids, pregnant or lactating individuals, and those with previously initiated or completed root canal treatments were excluded from the study.

Postoperative pain intensity was assessed in patients undergoing root canal treatment who received either 4 mg of oral dexamethasone (DEXA group) administered one hour prior to the procedure or 400 mg of oral ibuprofen (CTR group) administered immediately after treatment. Pain levels were measured at 4, 6, 8, 12, and 24 h postoperatively using the Visual Analogue Scale (VAS), a validated instrument for quantifying perceived pain intensity on a 0–10 scale. The VAS consists of a 10-cm horizontal line anchored by the descriptors “no pain” (0) on the left and “severe pain” (10) on the right. For interpretive consistency, VAS values were classified as follows: 0–1 indicating no pain, 2–4 mild pain, 5–7 moderate pain, and 8–9 severe pain.

Dexamethasone, like other corticosteroids, provides potent anti-inflammatory and analgesic effects by suppressing the production and activity of multiple pro-inflammatory mediators responsible for pulpal and periapical inflammation [13]. Because its mechanism acts at the earliest stages of the inflammatory cascade, dexamethasone serves as an especially effective agent for preemptive analgesia, mitigating peripheral sensitization before it becomes clinically significant. The evidence presented here supports the utilization of single-dose preoperative corticosteroid therapy as an evidence-based strategy for managing post-endodontic pain, particularly in patients experiencing symptomatic inflammatory conditions. Its application should be individualized, taking into account patient health status and specific clinical indications.

Dexamethasone is widely regarded as safe when used in accordance with recognized clinical standards; however, its therapeutic benefits depend on variables such as dose, administration route, treatment duration, and patient-specific factors. In this study, oral administration was selected for its practical advantages in outpatient dental care. Oral dosing enhances patient comfort, improves compliance, and avoids complications associated with injectable delivery methods. This non-invasive approach also simplifies clinical workflow and reduces procedural demands.

Although single-dose corticosteroid therapy is generally safe, careful adherence to the prescribed dosage is essential, given the potential systemic risks associated with repeated or prolonged use. When employed judiciously, dexamethasone offers a highly favorable benefit–risk profile, delivering effective analgesia while maintaining a strong safety margin.

The evidence obtained consistently indicates a lower perception of postoperative pain in the group that received dexamethasone preoperatively compared to the control group treated with ibuprofen postoperatively. The analgesic effect of dexamethasone was evident across all measured time intervals, suggesting its efficacy in preemptively modulating the inflammatory cascade associated with root canal treatment. Importantly, no adverse effects were reported in association with either medication throughout the study period.

The results of the present study align with previous findings in the literature regarding the efficacy of dexamethasone in controlling postoperative endodontic pain. Nogueira et al. [14] concluded that the administration of 4 mg of dexamethasone significantly reduced postoperative discomfort compared to placebo, highlighting the advantage of its prolonged biological half-life ranging from 36 to 72 h as a long-acting corticosteroid. Similarly, Glassman et al. [15] demonstrated that systemic administration of dexamethasone (three 4 mg tablets) produced a statistically significant reduction in pain intensity at 48 h post-procedure (p < 0.01). In that study, the corticosteroid was administered both one hour before and four hours after endodontic treatment, though it was compared exclusively to a placebo group.

In contrast, our study employed a simplified and clinically practical protocol: a single oral dose of 4 mg dexamethasone administered one hour prior to the procedure. This was directly compared with a control group receiving 400 mg of ibuprofen postoperatively. The findings revealed that preemptive administration of dexamethasone resulted in a more substantial reduction in postoperative pain intensity than the standard NSAID protocol. This supports the hypothesis that preoperative corticosteroid therapy can more effectively suppress the early inflammatory response by modulating the release of pro-inflammatory mediators, thereby mitigating peripheral sensitization and improving patient comfort during the initial recovery phase.

Furthermore, the use of a single preoperative dose of dexamethasone minimizes the potential for adverse systemic effects commonly associated with prolonged corticosteroid therapy. As noted by Mehrvarzfar et al. (2008), although concerns remain regarding possible immunosuppressive effects of glucocorticoids such as increased susceptibility to infection the literature suggests that a single dose does not result in clinically significant harm [16]. Therefore, a single-dose premedication strategy with dexamethasone appears to offer an effective and safe option for the management of endodontic pain, particularly in otherwise healthy individuals.

Upon detailed review of the current literature, the only recent randomized clinical trial specifically evaluating pharmacologic agents administered during the preoperative phase of the root canal treatment was conducted by Kongala et al. in 2019 [17] this study investigated the comparative efficacy of a single oral dose of piroxicam, dexamethasone, and deflazacort as premedication for managing postoperative endodontic pain following root canal instrumentation.

The trial assessed the incidence and intensity of postoperative pain in teeth with both vital and non-vital pulps after biomechanical preparation. It was reported that the administration of 4 mg dexamethasone resulted in a reduction in pain levels comparable to that achieved with 20 mg piroxicam and 30 mg deflazacort. While the prophylactic administration of 20 mg piroxicam was found to be slightly more effective than deflazacort in reducing postoperative discomfort, dexamethasone consistently demonstrated superior pain reduction at all evaluated time intervals. However, the study concluded that the differences among the three pharmacologic agents were not statistically significant.

Cumulative evidence from this trial underscores the potential utility of corticosteroids such as dexamethasone in preemptive analgesic protocols for endodontic procedures. Although the comparative analgesic effects among dexamethasone, piroxicam, and deflazacort did not reach statistical significance, the trend toward greater pain reduction with dexamethasone aligns with our own results, further supporting its clinical relevance in managing endodontic pain through single-dose preoperative administration.

### This study is not without limitations, which should be acknowledged when interpreting the results

#### Regulatory constraints in Spain

In Spain, pharmaceutical use and clinical research are subject to stringent regulatory oversight. All experimental or investigational studies involving drug administration must be approved by an accredited ethical review board a process that can be prolonged and may pose logistical challenges in initiating and conducting time-sensitive clinical trials.

#### Patient adherence and data accuracy

Although patients were thoroughly instructed on the use of the Visual Analogue Scale (VAS) and the specific time points for recording pain intensity, there remains inherent uncertainty as to whether all participants adhered precisely to the prescribed timeline and evaluation protocol. Variations in adherence may introduce a degree of reporting bias, potentially affecting the reliability of the pain assessments.

#### Limited contemporary comparative evidence

The availability of recent, high-quality clinical studies examining preoperative pharmacologic strategies for endodontic pain management is limited. As a result, there are constraints in verifying or contextualizing our findings against a broader and more updated body of evidence. This underscores the need for continued research in this area to establish more robust clinical guidelines.

## Conclusion

Preoperative administration of a single 4 mg oral dose of dexamethasone provides a clinically and statistically superior reduction in postoperative pain compared with conventional postoperative ibuprofen therapy in single-visit root canal treatment. Dexamethasone achieved consistently lower VAS pain scores across all time points without adverse events or the need for rescue analgesia, underscoring its safety and robust analgesic performance. These findings substantiate the efficacy of low-dose preemptive corticosteroid therapy as a reliable strategy for optimizing postoperative pain control in endodontics and justify its consideration for integration into evidence-based clinical practice. Further large-scale, multicenter trials are recommended to validate and generalize these results.

## Supplementary Information

Below is the link to the electronic supplementary material.

Supplementary file1 (DOCX 48 kb)

## Data Availability

The datasets generated and analyzed during the current study are available from the corresponding author upon reasonable request. De-identified individual participant data (IPD) and supporting clinical documents will be made accessible in accordance with data protection and institutional policies.
