# Supplementary material for: Therapeutic management of dexamethasone in patients undergoing endodontics: a randomized clinical trial
Source: Odontology. 2025 Dec 19;114(4):2158–69. doi: 10.1007/s10266-025-01277-0 (PMC13630946; doi:10.1007/s10266-025-01277-0)
Supplement: Supplementary file 1 [file 10266_2025_1277_MOESM1_ESM.docx]

**Supplementary Figure 1**: **Consort 2010 checklist of information to include when reporting a randomised clinical trial.**

**CONSORT 2010 checklist of information to include when reporting a randomised trial***

| **Section/Topic** | **Item No** | **Checklist item** | **Reported on page No** |
| --- | --- | --- | --- |
| **Title and abstract** | | | |
|  | 1a | Identification as a randomised trial in the title | 1 |
|  | 1b | Structured summary of trial design, methods, results, and conclusions (for specific guidance see CONSORT for abstracts) | 1 |
| **Introduction** | | | |
| Background and objectives | 2a | Scientific background and explanation of rationale | 1 |
|  | 2b | Specific objectives or hypotheses | 2 |
| **Methods** | | | |
| Trial design | 3a | Description of trial design (such as parallel, factorial) including allocation ratio | 3 |
|  | 3b | Important changes to methods after trial commencement (such as eligibility criteria), with reasons | 3-4 |
| Participants | 4a | Eligibility criteria for participants | 4 |
|  | 4b | Settings and locations where the data were collected | 3-5 |
| Interventions | 5 | The interventions for each group with sufficient details to allow replication, including how and when they were actually administered | 5-6 |
| Outcomes | 6a | Completely defined pre-specified primary and secondary outcome measures, including how and when they were assessed | 5-6 |
|  | 6b | Any changes to trial outcomes after the trial commenced, with reasons |  |
| Sample size | 7a | How sample size was determined | 6 |
|  | 7b | When applicable, explanation of any interim analyses and stopping guidelines |  |
| Randomisation: |  |  |  |
| Sequence generation | 8a | Method used to generate the random allocation sequence | 3-4 |
|  | 8b | Type of randomisation; details of any restriction (such as blocking and block size) |  |
| Allocation concealment mechanism | 9 | Mechanism used to implement the random allocation sequence (such as sequentially numbered containers), describing any steps taken to conceal the sequence until interventions were assigned | 3-6 |
| Implementation | 10 | Who generated the random allocation sequence, who enrolled participants, and who assigned participants to interventions |  |
| Blinding | 11a | If done, who was blinded after assignment to interventions (for example, participants, care providers, those assessing outcomes) and how |  |
|  | 11b | If relevant, description of the similarity of interventions |  |
| Statistical methods | 12a | Statistical methods used to compare groups for primary and secondary outcomes | 6 |
|  | 12b | Methods for additional analyses, such as subgroup analyses and adjusted analyses |  |
| **Results** | | | |
| Participant flow (a diagram is strongly recommended) | 13a | For each group, the numbers of participants who were randomly assigned, received intended treatment, and were analysed for the primary outcome |  |
|  | 13b | For each group, losses and exclusions after randomisation, together with reasons |  |
| Recruitment | 14a | Dates defining the periods of recruitment and follow-up | 3-5 |
|  | 14b | Why the trial ended or was stopped |  |
| Baseline data | 15 | A table showing baseline demographic and clinical characteristics for each group |  |
| Numbers analysed | 16 | For each group, number of participants (denominator) included in each analysis and whether the analysis was by original assigned groups |  |
| Outcomes and estimation | 17a | For each primary and secondary outcome, results for each group, and the estimated effect size and its precision (such as 95% confidence interval) |  |
|  | 17b | For binary outcomes, presentation of both absolute and relative effect sizes is recommended |  |
| Ancillary analyses | 18 | Results of any other analyses performed, including subgroup analyses and adjusted analyses, distinguishing pre-specified from exploratory | 7-8 |
| Harms | 19 | All-important harms or unintended effects in each group (for specific guidance see CONSORT for harms) |  |
| **Discussion** | | | |
| Limitations | 20 | Trial limitations, addressing sources of potential bias, imprecision, and, if relevant, multiplicity of analyses | 9-10 |
| Generalisability | 21 | Generalisability (external validity, applicability) of the trial findings |  |
| Interpretation | 22 | Interpretation consistent with results, balancing benefits and harms, and considering other relevant evidence | 9-10 |
| **Other information** | | |  |
| Registration | 23 | Registration number and name of trial registry | 3 |
| Protocol | 24 | Where the full trial protocol can be accessed, if available | 3 |
| Funding | 25 | Sources of funding and other support (such as supply of drugs), role of funders |  |

**Supplementary Figure 2: Likert-type Visual Analogue Scale (VAS) questionnaire.**

**Visual Analog Scale (VAS) questionnaire for Pain.**

**University of Salamanca.**

**Study Title:** Therapeutic management of Dexamethasone in patients undergoing endodontics.

**Chief investigator:** Javier Flores-Fraile. University of Salamanca. Email: j.flores@usal.es

**Principal investigator**: Heilyn Joanna Nils. University of Salamanca. Email: hjnils@outlook.com

**Principal investigator:** Cristina Arce Recatalá. Valencia International University. Email: cristina.arce@professor.universidadviu.com

**Principal investigator:** Cosimo Galletti. Kore University of Enna, 94100 Enna, Italy; cosimo.galletti@unikore.it

**Objective of the Study:**

You are invited to participate in a clinical research study. The objective of the present study is to evaluate the anti-inflammatory efficacy of the preoperative administration of dexamethasone, 4 mg, orally, to control postoperative pain in endodontic intervention in any dental group, whether maxillary or mandibular, in a single visit.

**Instructions:**

Please indicate the intensity of your pain at 4,6,8,12, 24h after your post-operatory treatment by placing on the line below at the point that best represents your pain level. The line represents a continuum from No Pain to Worst Pain Imaginable. The left end of the line is anchored with a score of 0, indicating the complete absence of pain. Conversely, the right end is anchored with a score of 10, representing the worst pain imaginable

**Post-operative Pain Intensity Scale:**

Do you need any drug to control the pain? Which one and doses? _______________________

Location of the Pain___________________________

Duration of the Pain: _______________________________

Patient Information:

Name: _________________

Age__________________

Gender: __________________

Patient Signature:

I hereby confirm that the above information accurately represents my current pain level

________________________

**Supplementary Figure 3: Informed Consent and Authorization Form for Participation in the Clinical Trial.**

**Study Title:** Therapeutic management of Dexamethasone in patients undergoing endodontics.

University of Salamanca.

**Chief investigator:** Javier Flores-Fraile. University of Salamanca. Email: j.flores@usal.es

**Principal investigator:** Heilyn Joanna Nils. University of Salamanca. Email: hjnils@outlook.com

**Principal investigator:** Cristina Arce Recatalá. Valencia International University. Email: cristina.arce@professor.universidadviu.com

**Principal investigator:** Cosimo Galletti. Kore University of Enna, 94100 Enna, Italy; cosimo.galletti@unikore.it

**Objective of the Study:**

You are invited to participate in a clinical research study. The objective of the present study is to evaluate the anti-inflammatory efficacy of the preoperative administration of dexamethasone, 4 mg, orally, to control postoperative pain in endodontic intervention in any dental group, whether maxillary or mandibular, in a single visit.

**Procedures:**

If you agree to participate, you will be asked to answer the questionnaire

**Confidentiality:**

Your personal information and any data collected during this study will be kept confidential. Data will be stored securely, and only authorized investigators will have access. Results may be published, but they will not include any information that could identify you.

**Contact information:**

If you have any questions or concerns about the study, or if you experience any discomfort, please contact Heilyn Nils (973)4582473/email: [hjnils@outlook.com](mailto:hjnils@outlook.com)

**Authorization:**

By signing this form, you authorize the use and disclosure of your health information for the purposes of this study. This authorization is effective until the study is completed or until you withdraw your consent.

**Consent:**

I have read and understand the information provide above. I have had the opportunity to ask questions and have received satisfactory answers. I voluntarily agree to participate in this study and authorize the use of my heath information as described above.

Participant´s Name___________________________________________

Participant´s Signature________________________________________

Date______________________________________________________

Principal investigator´s Signature______________________________

Date___________________________________________________
